# Impact of early ambulation on functionality in patients undergoing valve replacement surgery

**Published:** 2021-11-06

**Authors:** André Luiz Lisboa Cordeiro, Jaildes Reis Dos Reis, Huendy Borges Da Cruz, André Raimundo Guimarães, Giulliano Gardenghi

**Affiliations:** ^1^Centro Universitário Nobre, Feira de Santana, Bahia, Brazil; ^2^Escola Bahiana de Medicina e Saúde Pública, Salvador, Bahia, Brazil; ^3^Instituto Nobre de Cardiologia, Feira de Santana, Bahia, Brazil; ^4^Hospital Encore, Aparecida de Goiânia, Goiás, Brazil

**Keywords:** early ambulation, heart valve prosthesis, intensive care unit, physiotherapy, thoracic surgery, walking

## Abstract

**Background:**

Cardiac surgery is a highly complex procedure that aims to increase and prolong the quality of life of patients. The role of physiotherapy in early mobilization after cardiac surgery has shown several benefits to the patient when he presents impairments in terms of functionality.

**Aim:**

To evaluate the impact of early ambulation on the functionality of patients undergoing cardiac valve replacement surgery.

**Materials and Methods:**

Prospective cohort study in patients undergoing cardiac valve replacement surgery. Patients had their functionality assessed preoperatively using the Functional Independence Measurement (FIM) and Perme Intensive Care Unit (ICU) Mobility Score scales. At ICU discharge, they were divided into two groups: walking group (WG) and non-WG (NWG). At discharge, the two functional scales were reapplied in these patients. Pre- and postoperative values were assessed using the independent Student’s *t*-test. It was considered statistically significant when *P*<0.05.

**Results:**

One hundred and seventy patients were evaluated, 110 (65%) male, with a mean age of 48±2 years. In relation to Perme Score, the WG had a decrease of 11±2 and the NWG had a decrease of 13±2 (*P*=0.34). Regarding FIM, those who walked had a decrease of 27±3 against those who did not walk, which showed a reduction of 36±5, with a significance level of *P<*0.001.

**Conclusion:**

Based on the FIM data found, patients undergoing cardiac valve replacement surgery who underwent early mobilization had less decrease of functionality compared to patients who did not ambulate.

**Relevance for Patients:**

Based on this article, we can demonstrate that walking while still in the ICU environment favors less loss of functionality for patients after valve replacement surgery.

## 1. Introduction

Cardiac surgery is a highly complex procedure that aims to increase and prolong the quality of life of patients who need this type of intervention. Cardiovascular diseases represent one of the main causes of mortality worldwide [1]. According to DATASUS from January to June 2008, a total of 10,652 coronary artery bypass grafts and/or valve replacement surgeries were performed in Brazil [2]. Population aging, obesity, smoking, physical inactivity, and systemic arterial hypertension are important risk factors for heart disease [3].

Even with the whole process of evolution and technological advancement in surgical procedures, there are still countless factors involved in cardiac surgery that can negatively affect functionality. In this context, immobilism and/or prolonged bed rest in the postoperative period stand out as some of the main factors that generate several complications. Among them are loss of muscle strength, decreased cardiorespiratory capacity, physical deconditioning, as well as reduction of pulmonary function [4].

Due to changes that post-operatively occur in the body, it is necessary to measure the functionality of these patients both before and after surgery. For this purpose, the Functional Independence Measure (MIF) and Perme Intensive Care Unit (ICU) Mobility Score scales can be used [5-7].

The role of physiotherapy in early mobilization after cardiac surgery has shown several benefits to the patient when impairments occur in terms of functional capacity. Once again, immobility and prolonged bed rest may lead to reduced range of motion (ROM), loss of muscle strength, and others [8].

Physiotherapy will act, therefore, as preventive healthcare to avoid/minimize these postoperative complications. Its performance involves active kinesiotherapy, intra-unit ambulation, use of breathing pattern techniques, which acts on the functional health of individuals, favoring physical parameters such as gain in muscle mass and strength. In this way, it improves the ROM of the joints, body balance, increasing cardiorespiratory capacity, among others. Thus, it accelerates the performance in activities of daily living, favoring a faster hospital discharge process and, thereby, boosts the quality of life of patients [9,10].

The aim of this study was to assess the effects of early ambulation on the functionality of patients undergoing valve replacement.

## 2. Materials and Methods

This is a prospective cohort study carried out with the group of patients admitted to the Inpatient Unit at the Instituto Nobre de Cardiologia in Feira de Santana – BA from February 2015 to November 2019. The research was approved by the Research Ethics Committee of Faculdade Nobre, number 2,088,633, and all participants, after they were invited and agreed to participate in the study, signed the Free and Informed Consent Form before the study began.

### 2.1. Eligibility criteria

Male and female individuals aged 18 years or over who had undergone an elective valve replacement surgery (aortic and/or mitral) were included. Patients with cognitive impairment that prevented functional evaluation, death, or patients with more than four post-operative days in the ICU were excluded from the study.

### 2.2. Study protocol

All patients had their functionality assessed pre-operatively using the FIM and Perme ICU Mobility Score (Perme) scales. FIM was analyzed by separating the motor and cognitive domains. Both scales were applied by a blind examiner.

The day after the evaluation, patients were referred to the operating room and, later, to the ICU. At ICU discharge, they were divided into two groups: the walking group (WG), who walked at least 15 m in the ICU until discharge; and the non-WG (NWG) who did not walk or did the activity in a distance of <15 m. At the time of hospital discharge, the two functional scales were reapplied in these patients. The distance of 15 m was established because it was the average walk for these patients after the surgical procedure.

In addition to walking, the two groups performed procedures such as breathing exercises, cycle ergometry, active kinesiotherapy, and sitting exercises in the armchair.

### 2.3. Measuring instruments

FIM was developed in the 1980s by an American Physical Medicine and Rehabilitation Task Force. Created to measure the performance of patients in the performance of 18 tasks classified by subgroups regarding self-care, sphincter control, transfers, locomotion, communication, and social cognition, where it is scored on a scale ranging from 0 to 7 points, with 0 equivalent to total dependence and a maximum score of 126 points which is equivalent to the independent performance of tasks [5].

The Perme Score is a scale that objectively measures the mobility condition of the inpatient and has a score ranging from 0 to 32 points, divided into 15 items, grouped into the following categories: mental state, potential barriers to mobility, functional strength, bed mobility, transfers, walking aid devices and resistance measures. In it, the higher the score, the lower the need for assistance, therefore, the lowest score is indicative of low mobility and greater need for assistance [7].

### 2.4. Data analysis

For data analysis, the SPSS 20.0 software program was used. For normality, the Shapiro-Wilk test was used. Categorical variables such as age and comorbidities were assessed with the Chi-square test. Continuous data were expressed as mean and standard deviation. For assessing pre- and post-operative values the independent Student’s *t*-test was used. For intragroup analysis, the paired Student’s *t*-test was used, and the significance value adopted was *P*<0.05.

## 3. Results

**Figure 1:**
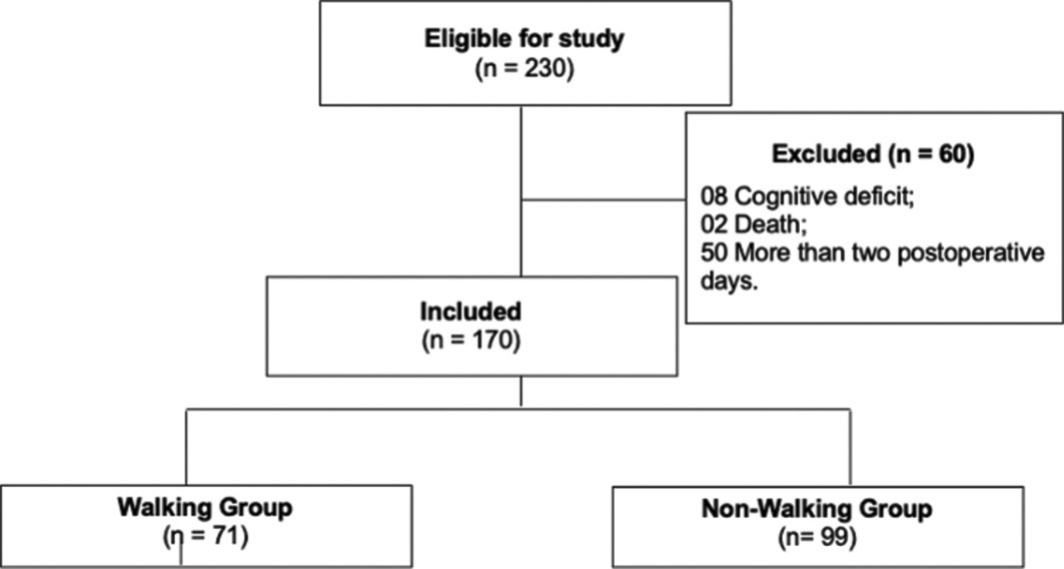
Steps for selecting the study subject

During the research period, 230 patients were hospitalized for heart valve replacement surgery, 60 patients were excluded from this total due to a cognitive deficit that prevented functional evaluation, death, or patients with more than two post-operative days (Figure 1).

Therefore, 170 patients were selected, 110 (65%) of whom were male, with mean age of 48±2 years, body mass index (BMI) 26±1 kg/m^2^. Systemic arterial hypertension was the main comorbidity as it was present in 39 (55 %) of these patients. The mean time for the first ambulation was 22±4 h. The other clinical and surgical data are shown in Table 1.

**Table 1:** Clinical and surgical data of the studied patients

Variable	Walking group (*n*=71)	Non-walking group (*n*=99)	Pa
Gender			0.14^a^
Male	49 (70)	61 (62)	
Female	22 (30)	38 (38)	
Age (years)	46±8	49±9	0.47^b^
BMI (kg/m^2^)	25±4	27±4	0.38^b^
Comorbidities			
SAH	39 (55)	46 (46)	0.23^a^
DM	21 (30)	29 (29)	0.69^a^
DLP	15 (21)	21 (21)	0.54^a^
Sedentary life	27 (38)	35 (35)	0.65^a^
CPB time (min)	62±8	65±9	0.26^b^
MV time (h)	6±3	7±4	0.68^b^
Grafts number	1±1	1±1	0.87^b^
ICU stay (days)	2±1	3±1	0.56^a^
Hospital stay (days)	7±2	8±1	0.63^a^
Surgery time (min)	198±34	205±41	0.34^b^
LVEF (%)	56±4	58±5	0.76^b^

a Chi-square;

b Independent Student’s T test; Gender and comorbidities variables are expressed as percentage. BMI: Body Mass Index; SAH: Systemic Arterial Hypertension; DM: Diabetes Mellitus; DLP: Dyslipidemia; CPB: Cardiopulmonary Bypass; MV: Mechanical ventilation; LVEF: Left ventricular ejection fraction; ICU: Intensive care unit.

Table 2 shows the data on the behavior of the intra and intergroup functionality studied. In the Perme scale, the WG had a decrease of 11±2 and the NWG had a decrease of 13±2 (*P*=0.34). In relation to FIM, those who walked had a decrease of 27±3 against those who did not walk, with a reduction of 36±5, with a significance level of *P*<0.001. The other values are shown in Table 3. The term ICU discharge refers to the postoperative moment.

**Table 2:** Behavior of the intra and intergroup functionality studied

Variable	Walking group (*n*=71)	Non-walking group (*n*=99)	*P* ^a^
Perme scale			
Preoperative	31±1	30±1	0.57
ICU Discharge	20±4	17±3	0.43
*P*^b^	0,03	0.04	
Delta	−11±2	−13±2	0.34
Hospital discharge	25±2	21±1	0.10
FIM total			
Preoperative	125±1	125±±1	0.92
ICU Discharge	98±4	89±7	<0.001
*P*^b^	<0.001	<0.001	
Delta	−27±3	−36±5	<0.001
Hospital Discharge	120±3	112±1	<0.001
FIM motor			
Preoperative	90±1	90±1	0.94
ICU Discharge	68±3	61±2	<0.001
*P*^b^	0,05	0,03	
Delta	−22±2	−29±3	<0.001
Hospital Discharge	88±3	81±3	<0.001
FIM cognitive			
Preoperative	35±1	35±1	0.89
ICU Discharge	30±2	28±2	0.23
*P*^b^	0.34	0.54	
Delta	−5±2	−7±2	0.42
Hospital Discharge	32±2	31±2	0.76

a Independent Student’s *t*-test; FIM: Functional Independence Measure; ICU: Intensive Care Unit.

**Table 3:** Adverse events verified during walking

Adverse events	*n*(%)
Lower limb pain	9(13)
Palpitation	7(10)
Dyspnea	4(6)
Dizziness	7(10)
Nausea	4(6)

We can see that there was a low incidence of adverse events in the WG (Table 3).

## 4. Discussion

In the present study, we found that early mobilization had an impact on functionality in patients undergoing valve replacement. Through the FIM it was observed that the WG lost less function than the NWG. Due to the specific characteristics associated with valve replacement, FIM seems to be more sensitive than Perme since the activities evaluated by FIM have a more severe impact on this population.

Possibly, the increase in respiratory capacity through responsible muscle activation stimulates the contraction of all peripheral muscles, increasing strength and preventing hypotrophy [11]. In line with the aforementioned, Ko *et al*. pointed out that early postoperative physiotherapy is increasingly recommended as it has many benefits in relation to muscle strength, physical conditioning, and health-related outcomes, that is, through walking, to enhance muscle activation, generating increased strength. In addition, it promotes better circulation that favors the transport of oxygen to the tissues, confirming that there may be improvement in terms of obtaining functionality, which positively agrees with the data obtained in our work. Such report shows that the intervention of the physiotherapist and the use of kinesio therapeutic techniques for the recovery and rehabilitation of patients is of paramount importance in the postoperative period [12].

It is worth noting that ambulation is part of the care protocol, that is, performing ambulation in isolation may not be enough to prevent the patient’s loss of function, which is confirmed by the study by Zanini *et al*. where they evaluated and demonstrated that the patients who performed ambulation together with other activities improved in terms of functional capacity, pulmonary function and muscle strength when compared to those who did only inspiratory muscle training or who did not undergo any intervention [13].

The approach of Hodgson *et al*. also suggested benefits regarding the effects of early mobilization. They emphasized that active kinesiotherapy, as well as walking associated with the use of new care protocols, such as cycle ergometry and electrical stimulation, for example, can provide improvement and restore strength. According to them, the intervention in the immediate post-operative period is essential, favoring the confirmation of the results presented in our work [14].

The study carried out by Almeida *et al*. also corroborated the present research as they approached physical exercise as a fundamental component in a cardiac rehabilitation program since it favors functional gains and, therefore, the kinesiotherapy protocols [15]. The authors also pointed out that active exercises have an effect on the stimulation of blood circulation, where, in addition to improving venous return, they favor gas exchange mechanism, intensifying the optimization of physical and cardiopulmonary capacity. These data reinforce the findings of the present research, raising the hypothesis that the need for physical therapy intervention results in the improvement of functional outcomes.

Results similar to our study were also found by Sarti, Vecina, Ferreira who stated that early physical therapy intervention prevents and decreases complications caused by immobilization. In addition, it optimizes recovery, significantly improving the functional dependence of patients, as well as decreasing the time of hospitalization, which automatically reduces costs for the unit [16].

Despite reports of delirium in the post-operative period of cardiac surgery, in our study, the motor domain of FIM was more affected. The presence of drains, a reduction in muscle strength and a decrease in lung function may be responsible for the worsening of the motor domain. The score of 36 points in patients who did not walk, which shows the worsening, can be associated with clinical and functional aspects as a limitation for performing activities after ICU discharge.

We also observed a reduction in functionality in the postoperative period when compared to the preoperative period. This reduction is due to the impact generated by the surgery and requires an early rehabilitation protocol. Showing a positive correlation with our study, Moradian *et al*. concluded from their research that immediate intervention, associated with other therapeutic measures, can be viable and safe, bringing benefits to the respiratory capacity, as well as to physical conditioning, improving muscle function [17]. Such factor can be explained by the influence of mobilization in increasing the production of oxygen to the cells, generating the necessary energy support for muscle contraction.

The studies in the literature focusing cardiac surgery corroborate the effectiveness of early mobilization. In line with the findings of the present study, Rocha *et al*. described that immobilism causes complications after cardiac surgery, which can be explained by the decrease in muscle strength, that is, these complications affect the integrity of the muscles and decrease functionality [18]. According to Truong *et al.*, the physiological mechanisms that can lead to this weakness and loss of muscle mass are related to decreased protein synthesis, indicating catabolism of this musculature, thus demonstrating that mobilization is essential and can improve strength, in addition to decreasing oxidative stress and promoting increased functionality, which may justify the findings of the present study [19].

In another study carried out by Monteleone *et al.*, prolonged rest is associated with negative outcomes, that is, the decline in the ability to walk, which may prevent progress in improving and recovering the patient. Therefore, an efficient treatment protocol is necessary, and mobilization is presented as a way to mitigate major complications. As it can be seen, this intervention is closely related to the benefits shown in the present study [20].

Further studies are required to determine the main barriers to early ambulation in patients after heart surgery. Furthermore, the relationship between early ambulation and long-term outcomes, including functional independence and health-related quality of life, has yet to be elucidated. The main study limitations were the use of a convenience sample, lack of randomization process, and a single-center recruitment.

## 5. Conclusion

Our results suggest that early ambulation seems to be linked with greater functional status at hospital discharge in patients undergoing valve replacement surgery.

### Conflict of Interest

The authors certify that they have NO affiliations with or involvement in any organization or entity with any financial interest (such as honoraria; educational grants; participation in speakers’ bureaus; membership, employment, consultancies, stock ownership, or other equity interest; and expert testimony or patent-licensing arrangements), or non-financial interest (such as personal or professional relationships, affiliations, knowledge, or beliefs) in the subject matter or materials discussed in this manuscript.
